# *Crmp4*-KO Mice as an Animal Model for Investigating Certain Phenotypes of Autism Spectrum Disorders

**DOI:** 10.3390/ijms20102485

**Published:** 2019-05-20

**Authors:** Ritsuko Ohtani-Kaneko

**Affiliations:** Graduate School of Life Sciences, Toyo University, 1-1-1 Itakura, Oura 374-0193, Japan; r-kaneko@toyo.jp

**Keywords:** collapsin response mediator protein 4, autism spectrum disorder, neurodevelopmental disorder, whole-exome sequencing, animal model, sex different phenotypes

## Abstract

Previous research has demonstrated that the collapsin response mediator protein (CRMP) family is involved in the formation of neural networks. A recent whole-exome sequencing study identified a de novo variant (S541Y) of collapsin response mediator protein 4 (CRMP4) in a male patient with autism spectrum disorder (ASD). In addition, *Crmp4*-knockout (KO) mice show some phenotypes similar to those observed in human patients with ASD. For example, compared with wild-type mice, *Crmp4*-KO mice exhibit impaired social interaction, abnormal sensory sensitivities, broader distribution of activated (c-Fos expressing) neurons, altered dendritic formation, and aberrant patterns of neural gene expressions, most of which have sex differences. This review summarizes current knowledge regarding the role of CRMP4 during brain development and discusses the possible contribution of CRMP4 deficiencies or abnormalities to the pathogenesis of ASD. *Crmp4*-KO mice represent an appropriate animal model for investigating the mechanisms underlying some ASD phenotypes, such as impaired social behavior, abnormal sensory sensitivities, and sex-based differences, and other neurodevelopmental disorders associated with sensory processing disorders.

## 1. Introduction

The formation of neural networks is temporally and spatially regulated by numerous molecules, such as extracellular molecules regulating cell adhesion and axon guidance, and intracellular signaling molecules regulating axon elongation and the formation of dendrites, spines, and synapses. Collapsin response mediator proteins (CRMPs) are intracellular signaling molecules elicited by extracellular signals (e.g., semaphorin (Sema) 3A and reelin) during neuronal migration, differentiation, neurite network organization, and even remodeling [1,2,3]. Genome-wide studies, genetic linkage analyses, proteomic analyses, and translational approaches have revealed altered expression levels of CRMPs in neurodevelopmental disorders, such as schizophrenia, attention-deficit/hyperactivity disorder (ADHD), and autism spectrum disorder (ASD) [3,4,5,6,7,8]. Similar findings have been observed for neurological disorders such as Alzheimer’s disease [9,10,11] and hyperalgesia syndrome [12,13,14,15]. Furthermore, during the past decade, many studies using knockout (KO) mice have demonstrated the role of CRMPs in the pathogenesis of neurodevelopmental disorders, as described in Section 3 below. In our recent whole-exome sequencing study, we identified a de novo variant of *CRMP4* in a male patient with ASD [8]. In this review, we discuss the functions of CRMP4 in the developing brain and the possible involvement of CRMP4 deficiencies and abnormalities in the pathogenesis of neurodevelopmental disorders.

## 2. Identification of CRMP4 

Sema1A guides the growth cone in the proper direction during neural circuit formation in the developing brain. Sema1A was first identified as fasciclin IV in *Drosophila* [16] and subsequently identified as collapsin in chickens [17]. Since then, numerous members of the Sema family have been identified. Among them, Sema3A has been implicated in each step of neural circuit formation from axonal and dendritic development to synaptic assembly [18,19,20,21]. Goshima et al. [22] identified a CRMP with a relative molecular mass of 62 kDa (CRMP-62), now known as CRMP2, which is required for Sema3A-induced inward currents in the *Xenopus laevis* oocyte expression system. The authors further reported that introduction of anti-CRMP-62 antibodies into dorsal root ganglion neurons blocks Sema3A-induced growth cone collapse [22]. In 1995, Minturn et al. identified a 64-kDa protein in the rat embryo known as turned on after division 64 (TOAD-64), which was eventually classified as CRMP4 [23,24]. The CRMP family comprises five homologous cytosolic proteins (CRMP1 ~ 5) with high (50−70%) homology. CRMP4 is also referred to as TUC-4, unc-33-like phosphoprotein 1 (Ulip-1), dihydropyrimidase 3 (DRP3), and dihydropyrimidase-like 3 (DPYSL3) because those were found to be homologous to CRMP4 later [23,24,25,26]. These multiple names of CRMP4 have sometimes caused confusion. 

## 3. The Regulatory Mechanisms Suggested for CRMP4

CRMPs regulate intercellular signaling pathways mediated through extracellular molecules such as Sema3A, reelin, neurotrophins, and myelin-associated inhibitors (MAIs) [22,23,24,25,26,27,28]. Through transduction of these extracellular cues, CRMPs have been reported to regulate various neurodevelopmental events including neuronal apoptosis, migration, axonal elongation, dendritic elongation and branching, spine development, and synaptic plasticity [27,28,29,30,31]. CRMP functions are controlled by the dynamic spatiotemporal regulation of phosphorylation status, which is mediated by kinases such as Cdk5, Rho/ROCK, and GSK3β, which alter CRMP binding to various cytoskeletal proteins such as actin, tubulin, and tau [32,33,34,35,36]. Cytoskeletal proteins regulate neuronal polarity, axonal and dendritic outgrowth, neuronal migration, synaptic formation, and other functions of neurons like transportation of neurotransmitters-containing vesicles. Therefore, effects on cytoskeletal dynamics promote neurodevelopmental responses mediated by CRMPs. 

Numerous studies have focused on the relationship between CRMP phosphorylation and the roles of CRMPs. For example, MAIs regulate neurite extension via the phosphorylation of CRMP4, which is mediated by upstream phospho-inactivation of GSK3β [28]. Loss of GSK3β phosphorylation permits L-CRMP4–RhoA binding and suppresses neurite outgrowth. Therefore, MAI−CRMP4 signaling normally contributes to myelin-dependent growth inhibition [37]. Additionally, phosphorylation of CRMP2 and CRMP4 by Cdk5 is required for the proper positioning of Rohon–Beard primary sensory neurons and neural crest cells as well as caudal primary motor neurons in the zebrafish spinal cord during neurulation [38,39]. 

In addition to phosphorylation, truncation of CRMP4 by calpain-mediated cleavage is found in glutamate- and N-methyl-D-aspartate (NMDA) receptor-induced excitotoxicity and oxidative stress, both of which reduce cellar viability in primary cultured cortical neurons [40,41,42]. The similar regulatory mechanism of CRMP4 is also involved in potassium deprivation-induced apoptosis in cultured cerebellar granule cells [43].

Furthermore, CRMP4 is expressed as both a short isoform (CRMP4a) and a longer isoform (CRMP4b) [44,45]. Previous studies have indicated that these two isoforms exhibit opposing functions during neurite outgrowth [44,46], though the mechanisms regulating their expressions remain unclear.

## 4. Potential Involvement of CRMPs Including CRMP4 in Neurodevelopmental Disorders

CRMP family genes and proteins are abundantly expressed in the developing brain, strongly suggesting that they play important roles in neuronal circuit formation [23,47,48]. Furthermore, in situ hybridization experiments have revealed that there are regional differences in *Crmp4* mRNA expression during postnatal brain development [49]. In addition, while *Crmp4* mRNA expression is scarcely detectable in most areas of the adult brain, it remains considerably detectable in adult neurogenic regions containing immature neurons, such as the subgranular zone of the dentate gyrus and subventricular zone–olfactory bulb (OB) migratory pathway [49]. Such findings highlight the crucial role of CRMP4 in neuronal circuit formation.

Abnormal CRMP expression in the brain has been associated with several neurodevelopmental disorders. For example, patients with schizophrenia exhibit alterations in levels of CRMP1 and CRMP2 protein (for review, see [4], [7,50,51,52]. Liu et al. [53] suggested that reduced transcription and mTOR-regulated translation of certain DPYSL2 isoforms (i.e., genes encoding CRMP2) increase the risk of schizophrenia. Lee et al. [6] further reported that two functional single-nucleotide polymorphisms of the human DRYSL2 gene are associated with susceptibility to schizophrenia. Pham et al. [7] demonstrated that allelic variants of the di-nucleotide repeat at the 5’-untranslated repeat of *DPYSL2* change the interaction between CRMP2 and mTOR effector proteins. In addition, findings obtained from *Crmp1*- and *Crmp2*-KO mice suggest that impairments in CRMP1 and CRMP2 functions are involved in the pathogenesis of schizophrenia [5,54,55]. Furthermore, brain-specific *Crmp2*-KO mice display molecular, cellular, structural, and behavioral deficits, many of which are reminiscent of the features associated with schizophrenia [56]. 

**Figure 1:**
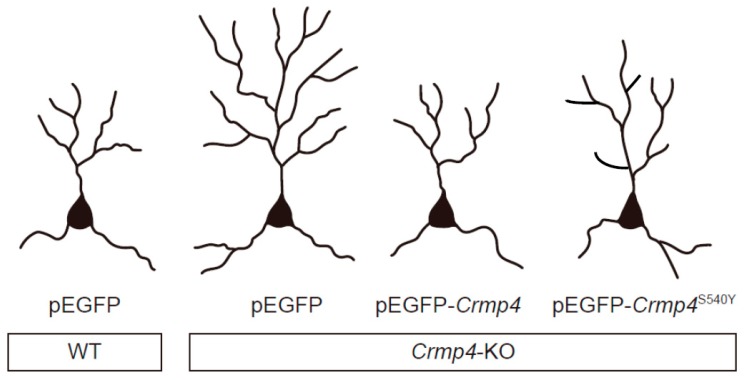
Schematic drawings showing dendritic arborization of cultured hippocampal pyramidal neurons differentially expressing CRMP4. The S540Y mutation in mouse *Crmp4* is homologous to S541Y in human *CRMP4*, which was observed in a patient with autism spectrum disorder (ASD). Representative drawings of cultured hippocampal cells from wildtype (WT) mice transfected with control (pEGFP) vector, *Crmp4*-knockout (KO) mice transfected with pEGFP vector, *Crmp4*-KO mice transfected with pEGFP-*Crmp4* vector, and *Crmp4*-KO mice transfected with pEGFP-*Crmp4*^S540Y^ vector. CRMP: collapsin response mediator protein.

In contrast to CRMP1 and CRMP2, relatively few studies have investigated the involvement of CRMP4 in neurodevelopmental disorders [8,57,58,59]. Miller et al. [57] suggested that microRNA (miR)-132, CREB-regulated miRNA associated with NMDAR signaling, is involved in the pathogenesis of schizophrenia and revealed that expressions of several genes including CRMP4 (DPYSL3) are regulated by miR-132, though the relation between CRMP4 and miR-132 and that between CRMP4 and schizophrenia remain unknown. A missense variant and four other de novo variants of the CRMP4 gene were identified in an ASD proband from the Simons Simplex Collection [58]. A recent whole-exome sequencing study also identified another likely pathogenic missense variant in the *CRMP4* gene (*CRMP4*^S541Y^) in a male patient with ASD [8]. In addition, Tsutiya et al. [8] investigated the effect of *Crmp4* missense mutation, which was found in ASD patients, on dendritic extension. In their study, dendritic formation was compared among neurons from wild type (WT) mice (WT neurons) transfected with enhanced green fluorescent protein (pEGFP), and neurons from *Crmp4*-KO mice (*Crmp4*-KO neurons) transfected with either a pEGFP, pEGFP-WT *Crmp4* or pEGFP-*Crmp4*^S540Y^ (the site homologous to human S541) (Figure 1). *Crmp4*-KO neurons transfected with pEGFP had significantly longer dendrites with more branching points than WT-neurons transfected with pEGFP. *Crmp4*-KO neurons transfected with pEGFP-*Crmp4*^S540Y^ exhibited significantly greater numbers of dendritic branching points than *Crmp4*-KO neurons transfected with pEGFP-WT *Crmp4* (Figure 1). These results suggest that ASD-linked CRMP4 mutations alter dendritic morphology. Furthermore, accumulating evidence suggests that *Crmp4*-KO mice exhibit several phenotypes that resemble those observed in human patients with ASD (DSM-V [60]). In the following sections, we review the autism-like phenotypes observed in *Crmp4*-KO mice and other animal models of ASD. 

## 5. Behavioral and Perceptual Abnormalities Observed in *Crmp4*-KO Mice

### 5.1. Impairments in Social Behavior

Tsutiya et al. [8,61] examined behavioral deficits in young or adolescent *Crmp4*-KO mice. Their open-field test and elevated plus maze results suggested that *Crmp4*-KO mice of both sexes exhibited locomotive activity and anxiety levels similar to those observed in WT mice. Similarly, the novel object recognition test revealed no significant differences in memory acquisition/retention between WT and *Crmp4*-KO mice of both sexes. However, the authors also utilized the three-chamber test for investigating social behavior, which compares time spent investigating (sniffing) a stranger mouse and a novel object. Male *Crmp4*-KO mice spent significantly more time in the “object side chamber” than in the “stranger side chamber”, while WT mice of both sexes and female *Crmp4*-KO mice spent more time sniffing the stranger mouse. In addition, in the social interaction test, male *Crmp4*-KO mice spent significantly less time actively interacting with a stranger mouse than male WT littermates, although there were no significant differences in the amount of active interaction between WT and *Crmp4*-KO females. These findings indicate that male-dominant impairments in social behavior can be observed in *Crmp4*-KO mice [8].

### 5.2. Abnormalities in Sensory Perception

“Hyper-reactivity or hypo-reactivity to sensory input” is among the diagnostic criteria for ASD specified in the DSM-V. Recent studies have indicated that patients with ASD exhibit neural hyperactivity [62,63], which may account for abnormal sensory sensitivity. Neuronal hyperactivity is considered to result from membrane hyperexcitability and/or abnormal connectivity in neural circuits, such as recurrent excitation or a change in the balance between excitatory and inhibitory synaptic input. Altered neural activity has also been observed in animal models of ASD. For example, mice with null mutations in the *Fmr1* gene exhibit social deficits [64] and impaired sensory adaptation [65], which may be due to cortical hyper-excitability [66]. In addition, mice with null mutations of *Shank2* exhibit social deficits [67,68] and have been reported to exhibit hypo-excitability to mechanical and noxious heat stimuli as well as to inflammatory and neuropathic pain [69]. 

Several studies have examined sensory perception in *Crmp4*-KO mice. Tsutiya et al. [8,61] reported that *Crmp4*-KO pups exhibit alterations in temperature and olfactory perceptions when compared to WT mice. Infant mice produce ultrasonic vocalizations (UVs) as a normal response to sensory stimulation [70], and the number of UVs is usually used to evaluate the sensory perception ability. 

The numbers of UVs emitted by WT and *Crmp4*-KO pups of both sexes are similar at room temperature (RT, 23 °C). However, when WT pups of both sexes and *Crmp4*-KO females were subjected to a 19 °C environment, they produced significantly more UVs. When moved from RT to 9 °C, both groups emitted significantly fewer UVs. Surprisingly, when *Crmp4*-KO males were moved from RT to a 19 °C environment, the authors observed no increases in the number of UVs emitted. However, the number of UVs significantly increased when *Crmp4*-KO males were moved from RT to 9 °C. These findings indicate that temperature perception markedly differs between *Crmp4*-KO males and WT mice of both sexes/*Crmp4*-KO females [8]. 

In addition, *Crmp4*-KO mice of both sexes demonstrate impaired olfactory sensitivity when compared to WTs [8,61]. In these previous studies, WT pups of both sexes produced more UVs during exposure to unfamiliar bedding than during exposure to familiar bedding. In contrast, there was no significant difference in the number of UVs emitted by *Crmp4*-KO pups of both sexes when exposed to the different smells.

Many people diagnosed with ASD have difficulty processing sensory information, which often manifests as hyper- or hypo-sensitivity to sensory stimuli. To examine whether *Crmp4* KO is associated with hyper- or hypo-sensitivity to olfactory stimuli, a previous study utilized immunohistochemical experiments to examine the expression of the neuronal activity marker c-Fos following exposure to the odorant ethyl acetate (EA) [61]. Cells positive for c-Fos were counted in each layer of the OB (glomerular layer (GL), external plexiform layer (EPL), mitral cell layer (MCL), granule cell layer (GCL)) and compared among male WT pups and *Crmp4*-KOs with or without EA exposure. In WT and *Crmp4*-KO males without EA stimulation, only a few c-Fos-positive cells were observed in sections of the OB. In accordance with a study by Van der Gucht et al. [71], who reported that c-Fos is expressed by neurons after sensory induction, many c-Fos-positive cells were detected after EA exposure. Research has indicated that specific odorants induce neuronal activity in a spatially restricted area known as the odorant map [72,73]. The study has reported that c-Fos-positive cells can be observed in restricted areas of the EPL, MCL, and GCL of WT pups after EA exposure, and that the distribution of these cells is similar for the previous work performed in adult WT mice exposed to EA [74]. In contrast, *Crmp4*-KO pups exhibited broad, dramatic increases in the number of c-Fos-positive cells in all OB layers following EA exposure. Therefore, the number of active neurons with c-Fos expression is much greater in *Crmp4*-KO pups than in WT pups after exposure to a single odorant (EA), suggesting that the altered olfactory perception observed in *Crmp4*-KO pups stems from neuronal hyperactivity in the OB and may be other brain areas related to perception. 

## 6. Altered Dendritic Arborization in *Crmp4*-KO Mice and *Crmp4*-Knockdown (KD) Neurons

As shown in Section 3, transfection of cultured hippocampal *Crmp4* −/− neurons with mutated mouse CRMP4^S540Y^ (homologous to human CRMP4^S541Y^ found in a patient with ASD) increases dendritic branching when compared to transfection of WT *Crmp4*. Several studies have also reported altered dendritic involvement in other mouse models of ASD [75,76,77,78]. Indeed, animal models of ASD exhibit hippocampal and cortical pyramidal neurons with significantly longer apical and basal dendrites, as well as significantly greater branching, than WT neurons.

Recent studies have reported that dendritic morphology and axon elongation are altered in *Crmp4*-KO mice and *Crmp4*-knockdown (KD) cells [46,59,79,80,81,82]. Niisato et al. [79,80] revealed that deficiency of CRMP4 increases the bifurcation of pyramidal neuron apical dendrites in the mouse hippocampus and in primary cultures. Cha et al. [81] further reported that overexpression of the C-terminal actin-interacting site of CRMP4 facilitates dendritic growth in cultured hippocampal neurons. Using the DiI tracing method, Tsutiya et al. [8] reported that the extension of apical dendrites from OB mitral cells in vivo is enhanced in *Crmp4*-KO neonates, to those in WT animals [59]. Exaggerated elongation of neurites has also been observed in hippocampal neuronal cell line (HT22) cells transfected with *Crmp4* siRNA (*Crmp4*-KD HT22 cells) [59]. In addition, dendritic length and branching are greater in cultured hippocampal pyramidal neurons derived from *Crmp4*-KO neonates than in those derived from WT mice [8]. In contrast, overexpression of *Crmp4* suppresses dendritic elongation and branching in these cells [8]. Collectively, these results suggest that deficiencies in CRMP4 can increase dendritic elongation and branching in various types of neurons.

## 7. Altered Expressions of Genes Related to Excitatory and Inhibitory Synaptic Transmission in the Brain of *Crmp4*-KO Mice

ASD has been reported to be associated with alterations in the expression of several genes related to receptors, transporters, and synthesis enzymes for neurotransmitters such as glutamate, γ-aminobutyric acid (GABA), dopamine, serotonin, acetylcholine, and histamine (for review, see [83]). In addition, *Crmp4*-KO mice exhibit alterations in the expression of genes mainly related to the glutamatergic and GABAergic systems [8,61]. Since abnormalities in glutamate and GABA have been hypothesized to underlie ASD symptoms, recent translational proton magnetic resonance spectroscopy (MRS) studies have investigated levels of glutamate and GABA in adult humans with ASD as well as rodent ASD models. Such studies have reported that glutamate concentrations in the striatum are decreased in human patients with ASD and some animal models, although no such alterations in GABA levels were observed [84]. Glutamatergic abnormalities are well known to occur in models of ASD (for review, see [85]): For example, proline-rich synapse-associated protein 1 (ProSAP1/Shank2)-KO mice exhibit early, region-specific upregulation of ionotropic glutamate receptors at the synapse [67]. In addition, telomerase reverse transcriptase-overexpressing (TERT transgenic, TERT-tg) mice exhibit male-specific autism-like behaviors, as well as increases in the expression of the NMDA receptor NR2A and NR2B subunits and AMPA receptor GluR1 and GluR2 subunits. TERT-tg mice also exhibit increases in vesicular glutamate transporter (vGluT) 1 levels in the prefrontal cortex [86]. Furthermore, research has indicated that glutamatergic modulators may aid in the treatment of ASD in humans [83,87] and animal models [85,88,89], supporting the notion that the glutamatergic system plays a role in ASD and ASD-like phenotypes.

Male *Crmp4*-KO pups exhibit significantly greater mRNA and protein expressions of AMPA receptor subunits GluR1 and GluR2 than their WT counterparts [61]. Adult *Crmp4*-KO mice also exhibit sex- and region-dependent differences in levels of *GluR1*, *GluR2*, v*GluT1*, *vGluT2*, *GABAAα1*, *GABAAγ2*, *GABAB receptor 1*, and vesicular GABA transporter expressions. However, no significant differences in the expression of other genes (e.g., serotonin transporter mRNA in the raphe nucleus and dopamine D2 receptors (D2Rs) in the cortex) are observed between *Crmp4*-KO and WT mice of either sex [8]. These data support the notion that *Crmp4* deficiency induces alterations in glutamatergic- and some GABAergic-associated genes, which may be associated with the pathogenesis of certain autism-like features in *Crmp4*-KO mice. 

Many studies have implicated altered excitatory (glutamatergic)/inhibitory (GABAergic) balance in the pathogenesis of ASD [90,91,92,93]. However, altered gene expressions in *Crmp4*-KO mice do not necessarily mean the excitatory/inhibitory balance. Future physiological investigations of *Crmp4*-KO mice may help to reveal the functional meaning of alterations in glutamatergic and GABAergic gene expressions, and whether such alterations are associated with autism-like phenotypes.

## 8. Sex-Specific Phenotypes Observed in *Crmp4*-KO Mice and Other Animal Models of ASD

ASD is more prevalent among boys than girls, and there are substantial sex-based differences in ASD phenotypes [94,95,96,97]. For example, male-biased differences have been observed in patients with ASD exhibiting mutations in genes that encode the synaptic cell adhesion protein neuroligin (*NLGN),* including *NLGN 3,* and *NLGN4X* [98,99]. Sex-based differences in autistic-like phenotypes have also been reported in animal models of ASD generated by exposure to chemicals or genetic manipulation [8,67,86,100,101,102,103]. For example, Schneider et al. [102] demonstrated that prenatal exposure to valproic acid (VPA), which is well known to induce autism-like phenotypes in rats or mice [104], induces some male-specific alterations in behavior and immunological function. Kim et al. [86,101] further revealed that rats exposed to VPA in utero exhibit male-specific alterations in social interactions, hyperactive behavior, and impaired postsynaptic development. Konopko et al. [103] reported sex-based differences in the induction of some exons of the brain-derived neurotrophic factor *(Bdnf)* gene in the brains of fetal mice exposed to VPA during the prenatal period, indicating that female sex may confer neuroprotection against ASD-like phenotypes. 

Some animal models of ASD developed by deleting genes found to be mutated or deficient in human patients with ASD exhibit sex-based differences in autism-like phenotypes. For example, UVs in response to brief separation from the mother are more prominent in female *Nlgn4*-KO pups than in their male counterparts [105]. In addition, adult *Shank2* −/− mice exhibit limited male-biased differences in the call rate and duration of UVs [100]. Tsutiya et al. [8] identified a rare *Crmp4* mutation in male patients with ASD. Furthermore, *Crmp4*-KO mice exhibit male-biased alterations in social behavior, sensory perception, and gene expression, as described in the preceding sections (Section 3, Section 4, Section 5 and Section 6). Iwakura et al. [106] further revealed that CRMP4 is among the candidate proteins involved in the sexual differentiation of the anteroventral periventricular nucleus (AVPV) in the preoptic area of the hypothalamus. The AVPV, which is known to regulate ovulatory cycles, is larger in females than in males. This difference is due to the effects of testosterone (T) secreted from the testes of perinatal males [107]. In our previous study, which involved proteomics analysis followed by real-time PCR analysis, we observed that CRMP4 and *Crmp4* mRNA expression in the AVPV is sex-dependent during the critical period of sexual differentiation. In addition, prenatal testosterone propionate (TP) treatment increased the expression of *Crmp4* mRNA during the critical period in females [106]. Moreover, the number of dopaminergic neurons in the nucleus was influenced by *Crmp4* deletion in females, suggesting that CRMP4 plays a sex-dependent role in the regulation of dopaminergic neuronal death or survival in this region [106]. However, the relationship between decreases in the number of dopaminergic neurons in the AVPV and ASD-like phenotypes in *Crmp4*-KO females remains uncertain.

Ferri et al. [108] argued that the male predominance of ASD may be associated with interactions between risk genes, which may not be sex-specific themselves, and sex-specific hormonal or immune-related pathways. Although several candidate hormones have been proposed, it has been suggested that androgens are associated with such differences because it is well known that natural secretion of androgens from the testes during the prenatal period contributes to the increased risk of ASD in males (i.e., prenatal sex steroid theory) [109,110,111,112,113,114]. Accumulating evidence has demonstrated the important role of prenatal androgens in many aspects of neural network formation through their effects on developing neurons and glia–neuron interactions [115,116]. In these studies, it is reported that prostaglandin and gonadal steroids including T and estradiol converted from T by aromatase influence synaptogenesis via their effects on developing glial cells, astrocytes and microglia. In an intensive post mortem study, Werling et al. [117] observed increased expression of astrocyte and microglia marker genes in the brains of male patients with ASD, suggesting that interactions between glial cells and neurons may be involved in sex-based differences in ASD phenotypes. Although the mechanisms underlying the pathogenesis of ASD and the target cells of androgens and/or estrogens converted from androgens remain unclear, perinatal androgen exposure may contribute to the sexually dimorphic pathophysiology of ASD. According to the prenatal sex steroid theory of autism, loss of the suppressive role of CRMP4 in *Crmp4*-KO mice in neuronal development may exaggerate the promotive effect of prenatal sex steroids, androgens and/or estrogens converted from androgens secreted from the testes, on neuronal network development, thereby resulting in male-biased ASD-like phenotypes. 

## 9. Conclusions

**Figure 2:**
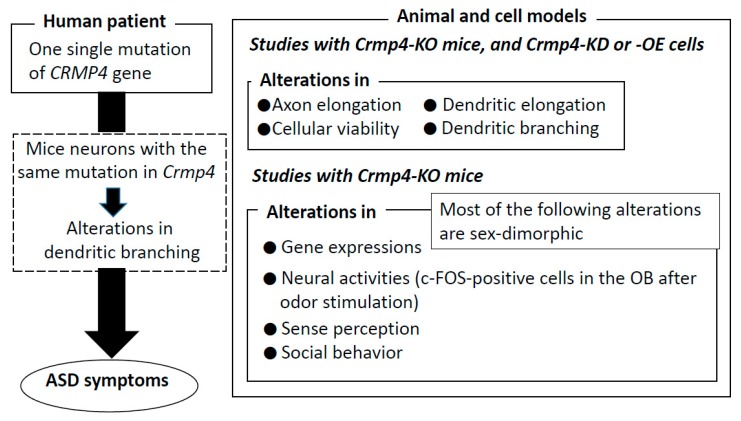
Summary of features associated with deficiency, overexpression, and mutation of *Crmp4*. *Crmp4*: collapsing response mediator protein; *Crmp4*-KO: *Crmp4*-knock out; *Crmp4*-KD: *Crmp4*-knockdown, *Crmp4*-OE: *Crmp4*-overexpression; OB: olfactory bulb; ASD: autism spectrum disorder.

CRMPs are known to regulate various aspect of neural development, playing key roles in neurodevelopmental disorders. As summarized in Figure 2, a previous whole-exosome sequencing study identified a single mutation of the *Crmp4* gene in a patient with ASD. Neurons from *Crmp4*-KO mice or neurons transfected with the mutation observed in the patient with ASD exhibit alterations in dendritic branching and/or extension (Figure 1). In addition, axonal elongation and cell viability are affected in *Crmp4*-KO mice, and in *Crmp4*-KD or -OE cells. *Crmp4*-KO mice also exhibit alterations in the expression of multiple genes contributing to glutamatergic and GABAergic neurotransmission, and most of these differences are sex- and region-specific. Single odorant stimulation induces hyperactivity (i.e., an increase in the number of c-Fos-positive cells) in the OB of *Crmp4*-KO pups. Furthermore, male *Crmp4*-KO mice exhibit more severe social and sensory deficits than females. Since most of their ASD-like phenotypes are sexually dimorphic, *Crmp4*-KO mice may represent a powerful model for investigating the pathogenesis of ASD and the prenatal sex steroid theory of autism in addition to *Crmp4*-KO mice possibly providing an animal model for investigating some other developmental disorders including ADHD and learning disabilities associated with sensory processing issues.

## Abbreviations

ADHD	attention-deficit/hyperactivity disorder
AMPA	α-amino-3-hydroxyl-5-methyl-4-isoxazole-propionate
ASD	autism spectrum disorder
AVPV	anteroventral periventricular nucleus
CRMP	collapsin response mediator protein
DRP	dihydropyrimidase; DPYSL3, dihydropyrimidase-like 3
EA	ethyl acetate
EPL	external plexiform layer
FMR1	fragile X mental retardation 1 gene
GCL	granule cell layer
GL	glomerular layer
GluR1	glutamate receptor 1
GluT1	glutamate transporter 1
KO	knockout
MAI	myelin-associated inhibitor
MCL	mitral cell layer
NMDA	N-methyl-D-aspartate
OB	olfactory bulb
RT	room temperature
TERT-tg mice	telomerase reverse transcriptase-overexpressing transgenic mice
TOAD-64	turned on after division 64
TUC-4	TOAD-64/Ulip-1/CRMP4
Ulip-1	Ulip-1
UV	ultrasonic vocalization
vGluT1	vesicular glutamate transporter 1
WT	wild type
